# Supplementary material for: Rationalizing the Dependence of Poly (Vinylidene Difluoride) (PVDF) Rheological Performance on the Nano-Silica
Source: Nanomaterials (Basel). 2023 Mar 18;13(6):1096. doi: 10.3390/nano13061096 (PMC10056420; doi:10.3390/nano13061096)
Supplement: Supplementary file 1 [file nanomaterials-13-01096-s001.zip › nanomaterials-2256377-supplementary.pdf]

Supplementary Materials

Rationalizing the Dependence of Poly (Vinylidene Difluoride) (PVDF) Rheological Performance on the Nano-Silica

Yi Cui [1,2,†], Yang Sui [1,2,†], Peng Wei [3], Yinan Lv [1,2], Chuanbo Cong [1,2], Xiaoyu Meng [1,2], Hai-Mu Ye [1,2] and Qiong Zhou [1,2,*]

1 Department of Materials Science and Engineering, New Energy and Material College, China University of Petroleum-Beijing, Beijing 102249, China
2 Beijing Key Laboratory of Failure, Corrosion, and Protection of Oil/Gas Facilities, China University of Petroleum-Beijing, Beijing 102249, China
3 State Key Laboratory of Tribology, Department of Mechanical Engineering, Tsinghua University, Beijing 100084, China
* Correspondence: zhouqiong@cup.edu.cn
† These authors contributed equally to this work.

S1: The peaks of 20.08° and 35.97° stand for the β-phase [1].

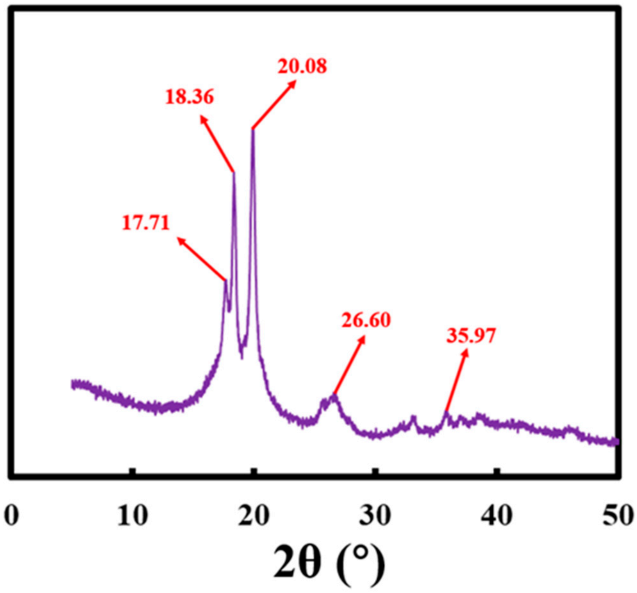

Figure S1. XRD pattern of pure PVDF.

Table S1. Conversion of mass fraction to volume fraction.

Mass fraction (wt%)	Mass of SiO_2 (g)	Volume of SiO_2 (cm^3)	Volume fraction (vol%)
0.1	0.01	0.090	1.59
0.2	0.02	0.181	3.13
0.3	0.03	0.273	4.63
0.4	0.04	0.364	6.08
0.5	0.05	0.455	7.49
0.6	0.06	0.545	8.85
0.7	0.07	0.636	10.17
0.8	0.08	0.727	11.46
0.9	0.09	0.818	12.71
1.0	0.1	0.909	13.93
2.0	0.2	1.818	24.45

The density of PVDF and nano-silica are 1.78 g/cm^3 and 0.11 g/cm^3. The volume fraction of nano-silica can be calculated as following:

$$Vol\ (\%) = \frac{V_{nano-silica}}{V_{PVDF} + V_{nano-silica}} \times 100\%$$

For all samples, the mass of PVDF is 10 g, so V_{PVDF} =10/1.78 = 5.618 cm^3

As the mass fraction of nano-silica raises from 0.1 wt% to 2.0 wt%, its volume fraction increases from 1.59 vol% to 24.25 vol%. The detailed result goes from one to the other can be seen in the Table S1.

S2: To confirm the presence of KH570 on the surface of the particles, infrared was performed on the nano-silica before and after modification. The FTIR spectrum in Figure S2 displays that the nano-silica after modification demonstrate the characteristic peaks of KH570, which are located at 1700, 2350, and 2960, while pure silica does not exhibit these peaks. Therefore, the nano-silica was successfully modified.

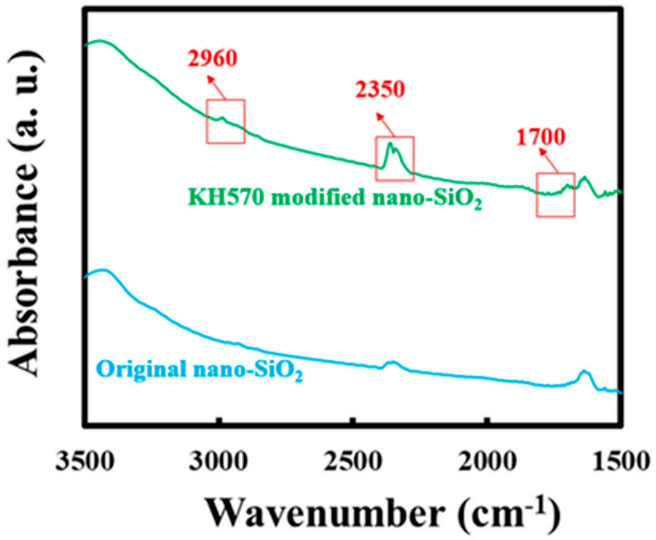

Figure S2. FTIR results of nano-silica before and after KH570 modification.

S3: According to the TEM images, the average diameter of 20-nm, 30-nm, and 50-nm modified nanoparticles is 19.85 ± 3.35 nm, 33.48 ± 5.64 nm, and 55.69 ± 4.97 nm, respectively. The specific analysis method is as following: TEM images were imported to ImageJ software. In ImageJ software, we set the size of scale bar firstly, and then measure each particle size by the measurement function of ImageJ (Figure S2). For each group of particles, we usually take about 30 TEM images, and measure at least 2–3 particle diameters in each image. A total of 50 particle diameters are measured, and then calculate the average value. Finally, the 50 diameter measurements were exported to Excel, and the average diameter of particles can be obtained.

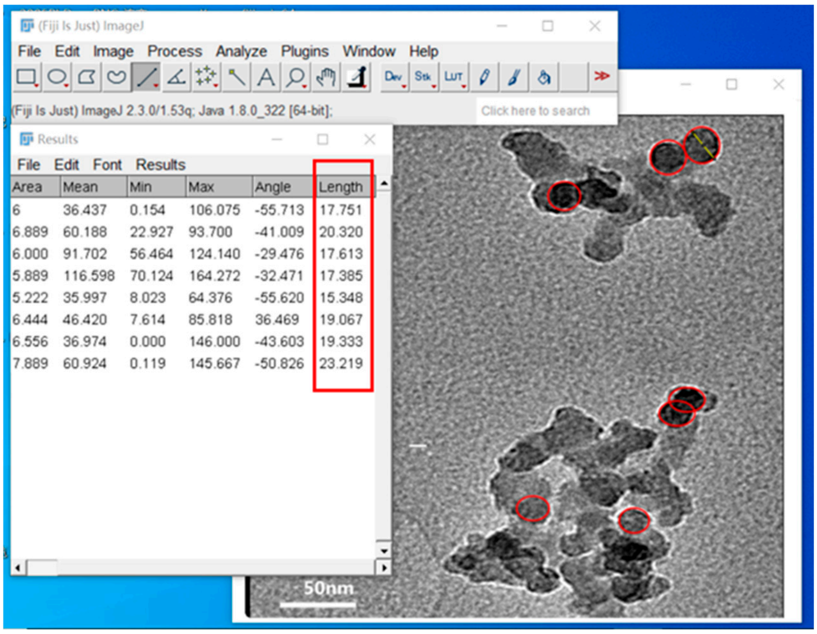

Figure S3. Schematic diagram of using ImageJ software to measure particle size.

S4: The calculations process of R_g

Figure 3 stands for the function graph of -ln I(q) vs q^2 and we can use Excel to fit the graph to get the slope and intercept.

In addition, we can obtain the equation $-\ln I(q) = \ln(\rho_0^2 v^2) + R_g^2 q^2/3$ from the equation $I(q) = \rho_0^2 v^2 e^{-\frac{q^2 R_g^2}{3}}$ (1). Then R_g of the sample can be calculated through the slope of linear fitting since the slope equals $R_g^2/3$.

For pure PVDF, the slope is 95.5418 ± 5.5706 nm, thus $R_g = \sqrt{3 \times 95.5418} = 16.93$ nm.

For PVDF/0.5 wt% TiO_2, the slope is 107.64 ± 6.7435 nm, thus $R_g = \sqrt{3 \times 107.64} = 17.97$ nm.

S5: Tan δ exhibits the minimum when the temperature is around 120℃.

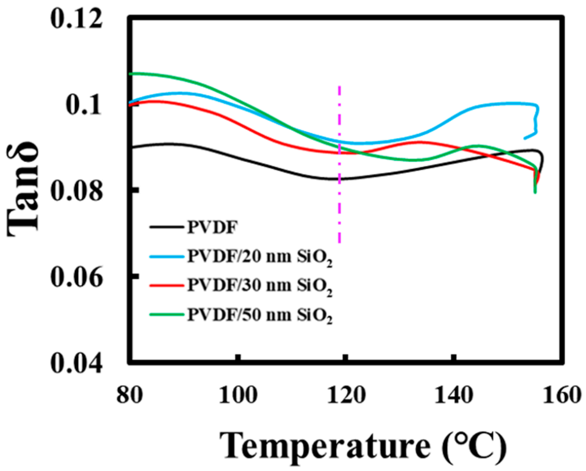

Figure S4. The changes of tanδ with temperature at 1Hz.

S6: The detailed calculation results of Me

The density is around 1.78 g/cm³; R equals 8.314 J/(mol • K); T is 393 K (120℃). Take the pure PVDF as an example to calculate the Me.

The plateau modulus of pure PVDF is 648.72 MPa, and thus:

$$\mathrm{Me} = \frac{1.78\ g/cm^3 * 8.314\ J/(mol \cdot k) * 393k}{648.72 * 10^6 Pa} = \frac{1.78\ g/cm^3 * 8.314\ J/(mol \cdot k) * 393k}{648.72 * 10^6 J/m^3} = 8.97\ \mathrm{g/mol}$$

$$\textbf{Me (PVDF/20 nm SiO}_2\textbf{)} = \frac{1.78\ g/cm^3 * 8.314\ J/(mol \cdot k) * 393k}{449.76 * 10^6 J/m^3} = \textbf{12.93 g/mol}$$

$$\textbf{Me (PVDF/30 nm SiO}_2\textbf{)} = \frac{1.78\ g/cm^3 * 8.314\ J/(mol \cdot k) * 393k}{441.78 * 10^6 J/m^3} = \textbf{13.16 g/mol}$$

$$\textbf{Me (PVDF/50 nm SiO}_2\textbf{)} = \frac{1.78\ g/cm^3 * 8.314\ J/(mol \cdot k) * 393k}{529.22 * 10^6 J/m^3} = \textbf{10.99 g/mol}$$

References:

1. Cao, X.; Ma, J.; Shi, X.; Ren, Z. Effect of TiO_2 Nanoparticle Size on the Performance of PVDF Membrane. *Appl. Surf. Sci.* **2006**, *253*, 2003-2010.
